# Editorial: Interoception, Contemplative Practice, and Health

**DOI:** 10.3389/fpsyg.2016.01898

**Published:** 2016-12-01

**Authors:** Norman Farb, Wolf E. Mehling

**Affiliations:** ^1^Department of Psychology, University of Toronto MississaugaToronto, ON, Canada; ^2^Department of Family and Community Medicine, and Osher Center for Integrative Medicine, University of California San FranciscoSan Francisco, CA, USA

**Keywords:** interoception, mindfulness, contemplative practice, consciousness, awareness

Well-being is deeply rooted in the body, a continuous flow of feelings denoting comfort or distress. Interoception, the representation of the body's internal state, is a growing target of scientific research, buoyed by a growing respect for contemplative traditions relating interoceptive awareness to the cultivation of well-being. An emerging interoception literature cuts across studies of neurophysiology, somatic anthropology, contemplative practice, and mind-body medicine. Key questions include: How is body awareness cultivated? What role does interoception play for emotion and cognition across the lifespan as well as in different psychopathologies? What are the neurophysiological effects of interoceptive training in Yoga, mindfulness meditation, Tai Chi, and other embodied contemplative practices? What categories from other traditions might be useful in this investigation? How might the cultivation of interoceptive awareness improve resilience in chronic health conditions?

Such questions have historically been ill-addressed by Western science, which is still influenced by a 400-year Cartesian tradition that treats cognition as something fundamentally distinct from sense-perception. This segregation limits consideration of interoception as the very context that situates and motivates cognition. And yet in therapeutic domains, attention to such context is critical, as the integration of interoceptive signals constrain the scope through which cognitive appraisals of well-being occur. This idea is not entirely novel to the literature- in 1974 Zanna and Cooper (1974) found elevated anxiety appraisals following caffeine pill ingestion when participants did not know they had consumed caffeine, as compared to when they did. Analogously, ignorance of the causes and consequences of our own myriad visceral feelings does not prevent them from coloring our interpretations of self, others, and the world. A strong version of this idea is that a failure to appreciate the causes and influences of interoceptive signals on cognition is to allow cognition to be determined by these signals.

It is still unknown how to comprehensively assess the quality of interoceptive integration. We know little about how to formally distinguish between adaptive and maladaptive interoception, or the consequences of dysregulated interoception on cognition and behavior. The complexity of these questions calls for the creative integration of perspectives and findings from related but often disparate research areas including clinical research, neuroscience, cognitive psychology, anthropology, religious/contemplative studies, and philosophy.

In recognition of the growing interest in such questions, we worked with Frontiers in Psychology to foster dialogue and collaboration in this emerging field. The numerous contributions stand as testament to a burgeoning interest in interoception, although direct demonstrations of interoceptive integration as a determinant of health were rare. Even rarer were empirical articles specifically addressing interoceptive reconditioning, in contemplative practice or beyond. Thus the role of interoceptive training in the context of wellbeing and health remains a relatively unexplored topic. Nevertheless, we are pleased to present this special issue that curates some of the growing interest in interoception research, speaking to the following research subdomains:

1. **Creating a scientific framework for our discourse**
  Farb et al. present an attempt at creating an integrated discourse on interoception from multiple perspectives, a “White Paper” on the theme of this special topic issue following a 2-day conference with researchers and scholars from Europe and the US from clinical and social psychology, medicine, psychiatry, neuroscience, nursing, and religion. The paper explores how contemporary theories of perception, e.g., the predictive coding model, can be applied to elucidate interoceptive processes, and mechanisms for reported health benefits of contemplative practice. It presents an overview of what we know today, the limitations and challenges we encounter in our field, and suggests next steps.
  Ceunen et al. review the history of interoception theory from its etymological origin through its subsequent semantic development. The authors trace the confusing language for the various components of interoception back to their physical and physiological contexts in which these were studied and discerned, such as by stimulus source, organs involved, and neurological signal-transmitting pathways, and propose a theoretical framework built around interoceptive experience understood as originating in the central nervous system.
  Schulz and Vögele review findings on psychobiological processes underlying acute and chronic stress and their interactions with interoception. They discuss psychological factors in stress reactivity, the role of the hypothalamic-pituitary-adrenocortical and the autonomic nervous system. The authors propose a theoretical model that integrates stress from early life and major adverse events with the dysregulation of physiological stress axes, altered interoception, the generation of physical symptoms, and the perpetuation of dysfunction.
2. **Interoceptive phenomenology across Eastern and Western cultural perspectives**
  Ma-Kellams explores the literature describing cross-cultural differences in interoception between Western and non-Western cultures and in levels of somatic awareness and interoceptive *accuracy*. She questions whether findings of heightened somatic awareness among non-Western cultures is linked to a greater emphasis on somatic symptoms in clinical depression and anxiety.
3. **Neural mechanisms underlying interoceptive processing**
  Wiebking and Northoff report an fMRI study of neural activity in the insula during the heartbeat counting task contrasting (a) non-psychiatric participants with high or low alexithymia scores to (b) patients suffering from major depressive disease and with comparable levels of alexithymia. The authors describe aberrant processing during an interoceptive task in depressed patients.
  Stoeckel et al. describe distinct brain activation patterns in dyspnea perception that indicate habituation or sensitization, a topic especially relevant for patients with bronchial and pulmonary diseases. They describe how trait and state anxiety and dyspnea unpleasantness are linked with specific patterns of brain activity and implicate the anterior insula and the periaqueductal gray regions in this context.
  Medford et al. studied physiological and neutral reactivity to emotion provocation in patients suffering from depersonalization disorder before and after pharmacological treatment. The authors relate reductions in symptoms central to this disorder, such as diminished sense of self and attenuated emotional experience, with normalization of neural responses to emotionally-evocative visual stimuli.
  Finally, focusing on brain structure rather than functional activity, Terasawa et al. studied the influence of insular damage on altered emotion processing and interoceptive awareness in three right insular and adjacent area-damaged individuals with well-preserved higher cognitive function. Emotion processing integrity was measured via recognition rates for emotional face stimuli, and examined for its association with interoceptive accuracy measured by the heartbeat perception task.
4. **Interoceptive awareness and contemplative practice**
  Bornemann et al. investigated how self-reported aspects of interoceptive awareness are influenced by a 3-month contemplative intervention that featured daily practices of attending to the body and breath. Interoceptive awareness was assessed by self-report with the German version of the Multidimensional Assessment of Interoceptive Awareness (MAIA). The authors address whether contemplative practices may target specific aspects of the complex multi-dimensional construct of interoceptive awareness.
  de Jong et al. report results of a randomized controlled trial of Mindfulness-Based Cognitive Therapy (MBCT) vs. usual care on self-reported interoceptive body awareness, assessed by the MAIA, in patients with chronic pain and comorbid depression. Mindfulness interventions including MBCT entail paying attention to present moment experience, including thoughts, emotions, and bodily sensations and train body awareness through the body scan and yoga. The study investigates whether MBCT produces selective and specific changes in interoceptive awareness, and whether such changes mediate improvements in clinical outcomes.
  Meditation reduces heart and respiration rates, blood pressure and skin conductance. Khalsa et al. explored the question whether meditators have stronger regulation skills for elevated cardiac adrenergic tone (heart rate; blood pressure) introduced by isoproterenol infusions compared to matched controls. Participants were exposed to different doses of isoproterenol or saline while resting or meditating.
  Is the subjective experience of the passing of time related to the awareness of body sensations, interoceptive accuracy and psychophysiological autonomic regulation? Otten et al. assessed performance in visual and auditory time duration reproduction tasks, comparing meditators with matched controls. They used the heartbeat perception task, self-reported presence and acceptance, the Attention Network Test, divided attention, heart rate, and skin conductance.
5. **Interoception as it relates to other psychological constructs and its role in clinical conditions**
  Ainley et al. examined a potential link between interoceptive accuracy and *empathy*. They compared the performance on the heartbeat perception task with scores on tests that relate to various aspects of empathy: Index of Interpersonal Reactivity, Questionnaire of Cognitive, and Affective Empathy, Reading the Mind in the Eyes Task for recognizing facial expressions involved in affect sharing, and Directors Task to assess cognitive perceptive taking ability involving self-other distinction.
  Cali et al. studied the complex relationship between distinct dimensions of self-reported interoceptive awareness and *emotional susceptibility*, the tendency to experience feelings of discomfort and vulnerability when facing negatively-valued emotionally-laden stimuli. They also investigated whether dimensions of self-reported interoceptive awareness, assessed by the MAIA, are related to interoceptive accuracy assessed by the heartbeat perception task.
  Longarzo et al. investigated the relationship between interoception and *alexithymia*, the difficulty to identify and express emotions cross-sectionally using the Toronto Alexithymia Scale and the newly developed Self-Awareness Questionnaire (SAQ) in healthy volunteers. The study explored whether alexithymia is related to interoceptive awareness, and in particular with a tendency to exaggerate fluctuations in body sensations.
  Pappens et al. paired a benign interoceptive stimulus as a conditioned stimulus (CS) with the unconditioned stimulus (US) of panic associated with feelings of suffocation—to study *fear learning* in healthy volunteers. Can the presentation of an interoceptive fear conditioning paradigm serve as a laboratory model for panic attacks? Conditioned fear learning was assessed by startle eye blink electromyography, skin conductance, and respiration rate and tidal volume. Is interoceptive fear learning independent of declarative knowledge of the CS–US contingency?
  Chan et al. explored the interoceptive processing of respiratory stimuli potentially altered by respiratory sensory gating in patients with generalized anxiety disorder compared to healthy controls. Respiration was manipulated through brief airway occlusions; sensory gating of stimulus processing was assessed by Respiratory-Related Evoked Potentials (RREP).
  Pollatos et al. report on two studies exploring whether higher levels of interoceptive sensitivity support the ability to overcome negative feelings caused by social exclusion in healthy volunteers. They created groups of high or low interoceptive performance using the heartbeat perception task. The experience of ostracism was induced by the cyberball paradigm, a computerized ball toss game in which the participant is eventually excluded from play. The first study assessed subjective feelings and behavioral affiliation tendencies, the second habitual emotion regulation processes focusing on suppression and reappraisal. Does interoceptive sensitivity reduce aversive states provoked by social exclusion and, if so, which coping strategy maximizes such protection?
  Payne et al. present a theoretical model that tries to understand the psychological and neurological dynamics of trauma, chronic stress, and post-traumatic stress disease, and how trauma therapy, specifically the Somatic Experiencing (SE™) approach, may rehabilitate functionally dysregulated autonomic, limbic, motor, and arousal systems. In a detailed case study, they illustrate the importance of taking into account the instinctive, bodily based protective reactions to stress and trauma and of using attention to interoceptive, proprioceptive, and kinesthetic sensation as a therapeutic tool.
  Quadro Motor Training (QMT) may be viewed as a radically simplified form of a mindful movement practice. In a 3-arm randomized controlled trial, Ben-Soussan et al. investigated the effect of four weeks of daily practice on cognitive flexibility and ideational fluency as dimensions of creativity, assessed with the Alternate Uses Task. They also followed three participants using structural MRIs and Diffusion Tensor Imaging to examine the relationship between gray matter volume, fractional anisotropy, and cognitive flexibility scores.
  Anxiety and anxiety sensitivity have been associated with increased interoceptive accuracy but also with bias for increased symptom reporting poorly correlated to physiological function. Petersen et al. attempted to disentangle interoceptive accuracy from bias by exposing groups of habitual high and low symptom reporters to a series of different inspiratory loads and asked participants for subsequent load classification. Signal detection analyses for classification accuracy were applied to differentiate interoceptive respiratory accuracy in symptom detection from classification bias. This is of clinical importance for asthma patients in the more ambivalent range of symptom intensity perceiving respiratory loads as either harmless or threatening.
6. **Interoceptive awareness in children**
  Georgiou et al. studied in children how interindividual differences in the perception of bodily processes, i.e., interoceptive sensibility/accuracy, based on heartbeat perception, interact with their degree of everyday physical activity measured by a multi-sensor device. Is higher interoceptive sensibility associated with stronger physical performance in children, which would indicate that interoception is important for the self-regulation of health-related behavior early in life.
  In a large prospective cohort study in Germany, Koch and Pollatos assessed interoceptive sensibility/accuracy in 1657 children (6–11 years old) twice 1 year apart and examined its prospective association with different food approach behaviors and weight status. They also determined whether altered interoceptive processes follow or precede non-adaptive eating behavior patterns in overweight children.
7. **Measuring subjective aspects of interoceptive awareness**
  Proven validity and reliability of measures for variations of interoceptive awareness and its multiple aspects are preconditions for informative research. Studies on objective, e.g., behavioral performance measures are urgently needed to move the research field forward. Regrettably, this topic issue did not receive any contributions for studies of objective assessment tools. However, several studies assessed the psychometric properties of systematically translated versions of the MAIA self-report measure. Bornemann et al. developed and studied the German version, Valenzuela-Moguillansky and Alejandro Reyes-Reyes the Spanish version, and Cali et al. the Italian version of the MAIA and assessed their psychometric properties in healthy volunteers. Taken together, these studies show strengths and weaknesses of the MAIA and provide suggestions for its further improvement. Longarzo et al. present the Self-Awareness Questionnaire (SAQ). Factor analysis of the SAQ showed two factors, related to visceral and to somatosensory sensations.
  This special issue serves only as a brief introduction to the growing field of research into interoception, embodiment, and somatic experience. The varied contributions in this issue serve to affirm the importance of interoception measurement and training for well-being and health. The psychometric studies herein helped to establish the multifaceted nature of interoception across a variety of cultural contexts and populations. Regarding quantitative measurements, heartbeat detection tasks seem to be effective in explaining variance in physical activity or symptom appraisal. And yet, this family of measures alone cannot possibly distinguish between the varied facets of interoception. Novel measures, such as the promising respiratory load task, are needed.
  While the initial returns on interoceptive awareness suggest that such awareness is likely beneficial in supporting well-being, it is also important for such inquiry to establish boundary conditions on the utility of enhancing awareness of interoceptive signals for particular people and in particular times. A central example of this problem lies in panic disorder, where those afflicted tend to demonstrate superior interoceptive accuracy on heartbeat detection compared to the general population (Ehlers, 1993). Thus, finding more nuanced methods to characterize what interoceptive signal is attended to, whether such attention is wisely timed, whether it serves to promote adaptive feelings and actions, and how variations in attention styles interact with interoceptive bias, all appear to be critical questions in continuing to develop an evidence-based account of interoception's role in determining health and well-being.
  Together, we hope these collected papers serve not only to validate the reader's belief in the importance of interoception for well-being across the lifespan, but also to stoke the imagination as to the unfolding mystery of how we come to know ourselves through our bodies. As these studies suggest, it is a rich time to join this field of research, with a need for new methods in measurement and training, in the hope of understanding the role of embodied feelings, and contemplative practices as contributors to well-being.

## Author contributions

NF and WM contributed equally to the manuscript.

### Conflict of interest statement

The authors declare that the research was conducted in the absence of any commercial or financial relationships that could be construed as a potential conflict of interest.
